# Real-world effectiveness of biologic therapies in severe asthma patients ineligible for phase 3 randomised controlled trials of biologics: an analysis from the UK Severe Asthma Registry

**DOI:** 10.1183/23120541.00565-2025

**Published:** 2026-01-19

**Authors:** Paul E. Pfeffer, Jola Karaj, Thomas Brown, Hassan Burhan, Rekha Chaudhuri, Kathryn Prior, Salman Siddiqui, Liam Heaney, David J. Jackson, Mitesh Patel, Pujan H. Patel, Hitasha Rupani, John Busby

**Affiliations:** 1 Barts Health NHS Trust, London, UK; 2 Barts and the London School of Medicine and Dentistry, Queen Mary University of London, London, UK; 3 Portsmouth Hospitals University NHS Trust, Portsmouth, UK; 4 Liverpool University Hospitals NHS Foundation Trust, Liverpool, UK; 5 Gartnavel General Hospital and University of Glasgow, Glasgow, UK; 6 Lancashire Teaching Hospitals NHS Foundation Trust, Lancashire, UK; 7 National Heart and Lung Institute, Imperial College, London, UK; 8 Queen's University, Belfast, UK; 9 School of Immunology and Microbial Sciences, King's College London, London, UK; 10 Guy's and St Thomas’ NHS Trust, London, UK; 11 Derriford Hospital, Plymouth, UK; 12 Royal Brompton Hospital, London, UK; 13 Imperial College, London, UK; 14 University Southampton NHS Foundation Trust, Southampton, UK; 15 Centre for Public Health, Queen's University, Belfast, UK

## Abstract

**Background:**

Most patients in real-world severe asthma populations would not be eligible for biologic randomised controlled trials (RCTs), although observational evidence has confirmed the effectiveness of biologics in real-world populations. We therefore investigated whether satisfying specific RCT inclusion/exclusion criteria affects biologic response in the real world.

**Methods:**

Inclusion and exclusion criteria from 11 pivotal phase 3 asthma biologics RCTs were reviewed to identify criteria themes, and median stringency within each characterised. Patients within the UK Severe Asthma Registry (UKSAR) with at least one year of follow-up on biologics were assessed as to whether they would satisfy inclusion/exclusion for each theme. Regression models were undertaken to assess whether the proportion of patients achieving a composite biologic response, defined as a ≥50% reduction in exacerbations or maintenance oral corticosteroids, was noninferior in patients ineligible by each theme. Superiority analyses and domain-specific responses were also analysed.

**Results:**

1421 adult patients with severe asthma from 13 specialist centres were included in this analysis. Noninferiority of composite response was demonstrated for all eligibility criteria except medication adherence. In superiority analyses, patients ineligible by adherence theme had a significantly lower odds ratio (OR) for composite response of 0.37 (95% CI 0.20–0.68), whilst patients ineligible by (low) baseline asthma symptom score had a significantly higher OR for response of 2.09 (1.31–3.32).

**Conclusions:**

Ineligibility by typical RCT inclusion/exclusion themes was generally not associated with inferior biologic composite response. Asthma biologics are effective in a broad range of patients, many of whom would not have met clinical trial eligibility criteria.

## Shareable abstract

**Severe asthma biologics are effective in a broad range of patients, including those not meeting the inclusion/exclusion criteria for phase 3 RCTs, although noninferiority could not be shown for those RCT-ineligible by poor medication adherence** https://bit.ly/40ZL8a8

## Introduction

Phase 3 randomised controlled trials (RCTs), required for drug licensing, have narrow inclusion and exclusion criteria to reduce heterogeneity in the clinical trial population and to actively exclude patients with characteristics that might adversely influence drug effect [1]. However, a consequence of these narrow criteria is that only a small proportion of patients with any disease are likely to be eligible for phase 3 clinical trials of treatment for that disease [1], an issue well-recognised in respiratory medicine [2]. Additionally, given the demands of frequent trial visits in many RCTs, such studies may inadvertently select patients with higher motivation to manage their disease and differing health behaviours. These issues raise the question of whether results from RCTs can always be generalised to real-world populations [1, 3]. Pragmatic trials and observational “real-world evidence” are therefore vital for the practice of evidence-based medicine and assessing benefits of new medications in broader patient populations [2].

Within severe asthma, an increasing number of biologics have become available with compelling RCT evidence that they reduce exacerbations, improve symptoms and limit oral corticosteroid (OCS) use. However, most patients being managed in specialist severe asthma centres would not qualify for the phase 3 RCTs that were conducted for licensing and approval of these biologic therapies [4, 5]. Nevertheless, observational and registry studies have shown real-world populations of patients with severe asthma to respond well to biologic therapies [5–8]. However, these analyses have only considered broad populations, and it remains to be seen whether specific patient subgroups, who were excluded from phase 3 RCTs, respond to biologics in real-world studies. For example, the comparatively poor clinical responses to many biologics in patients with the closely related pathology of COPD [9] raises the question of whether asthmatic patients with significant smoking history, who were excluded from the phase 3 RCTs in severe asthma, have adequate responses to biologics in the real world.

Biologics are high-cost therapies and as a result many healthcare systems restrict their use to patients most likely to benefit [10]. Given the resource implications, it is particularly important to know whether specific ineligibility criteria might identify patients who do not respond adequately to biologics. We chose a noninferiority design for the primary outcome in this study to better identify differences in response that would be both statistically and clinically significant [11], thereby potentially identifying patient groups who do not adequately respond and should not be treated with such biologics.

## Methods

### Identification of standard phase 3 RCT inclusion and exclusion criteria

The pivotal phase 3 RCTs for registration of the severe asthma biologics omalizumab, mepolizumab, benralizumab and dupilumab were identified through consensus discussion by the authors. Tezepelumab RCTs were not considered as this was not clinically available in UK at time of registry data capture. Eligibility criteria were extracted from the published manuscripts, including supplements, and where full criteria were not evident then from clinicaltrials.gov. Themes for inclusion and exclusion criteria were identified by the authors through consensus discussion, and for each theme the different specific criteria from the individual studies ranked from most stringent (most restrictive and limiting) to least stringent, and the wording of the criteria of median stringency noted. Inclusion criteria relating to background medication, T2 biomarkers and index exacerbation rate were not reviewed as UK approval for these biologics incorporates those criteria, with reimbursement criteria generally more restrictive than either the clinical trials’ eligibility for those criteria or reimbursement criteria in some other countries [10].

### Study population

The UK Severe Asthma Registry (UKSAR) collects data on patients with severe asthma referred to specialist services in the UK. Demographic characteristics, patient medical history, current medication, lung function and T2 inflammatory biomarkers are captured at baseline and response to treatment through annual follow-up entries [12]. Patients with data entry 2016–2023 were included in this analysis. The registry has ethical approval for collecting, storing, analysing and reporting such data with each patient's written consent (Office of Research Ethics Northern Ireland reference 15/NI/0196).

### Primary outcome

The primary outcome measure was a composite response to biologic, defined as a patient at follow-up achieving either 1) a ≥50% reduction in OCS requiring exacerbations without new initiation of maintenance OCS and/or 2) (where applicable) a ≥50% reduction in maintenance OCS (mOCS).

### Secondary outcomes

Additional domain-specific outcomes measures were assessed as secondary outcomes. Domain-specific responses analysed were daily symptom improvement, 6-item asthma control questionnaire (ACQ-6) improvement ≥0.5 or controlled (ACQ-6 <0.75); exacerbation reduction, >50% or no exacerbations; lung function improvement, forced expiratory volume in 1 s (FEV_1_) increase >100 mL; and reduced mOCS requirement, mOCS dose decrease ≥50% or no mOCS having been on mOCS at baseline.

### Statistical analysis

For the primary outcome a noninferiority analysis was chosen given the primary aim to ascertain whether any of the individual eligibility criteria were associated with a clinically significant reduction in biologic therapy response that could preclude future use of biologics in those patients. A noninferiority margin of 15% was chosen after consensus discussion. Assuming a biologic response rate of 80% for the primary outcome in eligible patients based on previous studies [13, 14], this corresponds to an OR (odds ratio) of 0.46 (*i.e.* the OR comparing an 80% response rate in the control group *versus* 65% response rate in the comparison group). The primary outcome of noninferiority was established if the 95% CI lower limit of the OR, comparing the probability of biologic response in the ineligible *versus* eligible group, was >0.46. A *post hoc* sample size calculation determined that eligibility criteria with a prevalence of at least 4.1% would provide 80% power to demonstrate noninferiority with a type 1 error of 0.025, *i.e.* that the smaller of the eligible and ineligible patient groups would need to include at least 4.1% of patients in whom that eligibility criteria can be assessed. Superiority analyses were conducted as secondary analyses.

We calculated descriptive statistics for demographic and clinical characteristics of UKSAR patients at baseline using means, medians and counts as appropriate. We conducted several logistic regression analyses investigating the association between each eligibility criteria and composite response. Minimally adjusted models included hospital site to prevent confounding by hospital referral criteria, and to account for the nonindependence of observations within hospitals. Fully adjusted models additionally included age (5-year categories), sex and pre-biologic blood eosinophil counts, factors independently associated with clinical response [15]. We choose this limited set of adjustment variables to prevent any overadjustment bias [16]. Similar models were fitted for domain-specific responses.

Observations with missing data were excluded from the analysis (supplementary table S1) without any attempt to impute values. All analyses were conducted using STATA version 16.

### Sensitivity analyses

Given the considerable overlap and at times diagnostic uncertainty between asthma and COPD, and separately with allergic-bronchopulmonary aspergillosis (ABPA), both exclusion criteria for asthma biologic phase 3 RCTs, we undertook sensitivity analyses for these comorbidities. Patients meeting standard, objective, diagnostic definitions for COPD and ABPA were identified in the UKSAR cohort, irrespective of whether the patients had been given those diagnostic labels, and we examined whether those patients had inferior responses to biologic treatment.

## Results

### Representative RCT inclusion/exclusion criteria

11 pivotal, phase 3 RCTs of biologics in severe asthma were identified through discussion between the collaborating researchers [17–27]. 10 themes for inclusion/exclusion criteria were initially identified; however, overlap between the themes of Comorbid Pulmonary Disease and Comorbid Other Eosinophilic Disease was noted, and therefore they were combined to generate nine common themes for phase 3 RCT asthma biologic inclusion/exclusion criteria (table 1, supplementary table S2). Within each theme the different specific inclusion/exclusion criteria for the 11 RCTs were ordered by stringency and the specific criteria of median stringency taken as representative of inclusion/exclusion criteria for that theme (table 1).

**TABLE 1:** Key themes for eligibility criteria for phase 3 randomised controlled trials (RCTs) of biologics in severe asthma, surrogate criteria in UKSAR and numbers of patients eligible/ineligible for each theme

Criteria theme	Median stringency RCT criterion	Representative operational criterion	Number of patients eligible^#^	Number of patients ineligible^#^
**Age**	ELIGIBILITY: Age 12 years−75 years inclusive	Age 12–75 years at baseline	1365 (96.1%)	56 (3.9%)
**Weight**	ELIGIBILITY: Weight ≥40 kg	Weight ≥40 kg at baseline	1409 (99.9%)	2 (0.1%)
**Confirmatory Diagnostic Lung Function**	ELIGIBILITY: Post-bronchodilator reversibility in FEV_1_ ≥12% and ≥200 mL, OR airway hyperresponsiveness with metacholine PC20 <8 mg·mL^−1^	Baseline post-bronchodilator reversibility in FEV_1_ ≥12% and ≥200 mL	238 (41.1%)	341 (58.9%)
**Impaired Lung Function**	ELIGIBILITY: Pre-bronchodilator FEV_1_ <80% predicted	Pre-bronchodilator/random FEV_1_ <80% predicted at baseline	848 (72.2%)	326 (27.8%)
**Uncontrolled Asthma Symptoms**	ELIGIBILITY: ACQ-6 score ≥1.5	ACQ-6 score ≥1.5 at baseline	1081 (84.9%)	192 (15.1%)
**Medication Adherence**	ELIGIBILITY: At least 70% compliance with usual asthma controller (ICS/LABA inhaler)	MPR for ICS ≥70% at baseline	915 (91.9%)	81 (8.1%)
**No Significant Smoking History**	INELIGIBILITY: Current smoker or ex-smoker with ≥10 pack-years smoking history	Current smoker or ex-smoker with ≥10 pack-years smoking history	1016 (74.0%)	357 (26.0%)
**No Substance Abuse**	INELIGIBILITY: History of alcohol or drug abuse within 12 months prior to the date informed consent, and assent when applicable, was obtained	Report in free-text comorbidity of any relevant comorbidities (see supplementary table 2)	1415 (99.6%)	6 (0.4%)
**No Comorbid Pulmonary/Other Eosinophilic Disease**	INELIGIBILITY: History of COPD, bronchiectasis, pulmonary fibrosis, cystic fibrosis, obesity hypoventilation, primary ciliary dyskinesia, α_1_-antitrypsin deficiency, lung cancer, ABPA/ABPM, EGPA, hypereosinophilic syndrome	Report in free-text comorbidity, other symptom and/or radiology descriptions of any relevant comorbidities (see supplementary table 2)	1039 (73.1%)	382 (26.9%)
**All Criteria (Fully Eligible)**	Not ineligible by any of the criteria		59 (15.2%)	330 (84.8%)

FEV_1_: forced expiratory volume in 1 s; ACQ-6: 6-item asthma control questionnaire; ICS: inhaled corticosteroid; LABA: long-acting β-agonist; MPR: medication possession ratio; ABPA: allergic bronchopulmonary aspergillosis; ABPM: allergic bronchopulmonary mycosis; EGPA: eosinophilic granulomatosis with polyangiitis. ^#^: number of UKSAR patients eligible/ineligible by each operational criterion included in these analyses.

### Clinical characteristics at baseline

To address the question of whether patients with severe asthma who would have been ineligible for phase 3 biologic RCTs have inferior responses to biologics, we analysed responses in a real-world cohort of patients from the UK Severe Asthma Registry. 1421 patients started on a biologic at baseline assessment and with annual follow-up review median (IQR) 404 days (364–495 days) after baseline assessment were included in this analysis (table 2, supplementary table S3 and figure S1). The mean age at baseline assessment was 51.6 years and the majority of patients were female (60.3%). The median number of exacerbations in the preceding year was 5 and 50.8% were on maintenance mOCS at baseline.

**TABLE 2:** Demographics of severe asthma patient cohort included in this analysis

Patients baseline characteristics	Entire cohort (N=1421)
**Age at first assessment years; n=1421**	51.6±14.7
**Age of onset years; n=1326**	26.1±20.0
**Sex; n=1421**	
Female	857 (60.3%)
Male	564 (39.7%)
**Ethnicity; n=1404**	
Caucasian	1186 (84.5%)
Non-Caucasian	218 (15.5%)
**BMI kg·m^−2^; n=1409**	30.7±7.4
**Smoking status; n=1393** ^#^	
Never smoked	911 (65.4%)
**Atopic disease; n=1401**	753 (53.7%)
**FEV_1_ L; n=1182**	2.03±0.80
**FEV_1_ % predicted; n=1174**	67.2±22.0
**FVC L; n=1167**	3.21±1.03
**FVC % predicted; n=1123**	85.2±19.6
**FEV_1_/FVC; n=1163**	63.3±18.3
**ACQ-6 score; n=1273**	2.8 (2.0–3.8)
**Uncontrolled asthma (ACQ-6 >1.5); n=1273**	1054 (82.8%)
**ACQ-7 score; n=1070**	3.0 (2.1–3.9)
**EuroQoL utility; n=714**	0.75 (0.51–0.89)
**Number of exacerbations (last year); n=1401**	5 (3–8)
**Any ED attendance (last year); n=1395**	530 (38.0%)
**Invasive ventilations (ever); n=1392**	134 (9.6%)
**Eczema; n=1421**	32 (2.3%)
**Nasal polyps; n=1421**	355 (25.0%)
**Gastro-oesophageal reflux; n=1421**	235 (16.5%)
**Depression or anxiety; n=1421**	125 (8.8%)
**Blood eosinophil count (×10^9^ L^−1^); n=1402**	0.38 (0.20–0.60)
**Highest blood eosinophil count (×10^9^ L^−1^); n=1395**	0.70 (0.43–1.10)
***F*_ENO_ ppb; n=1137**	44 (24–77)
**IgE IU·mL^−1^; n=1351**	160 (57–461)
**Maintenance OCS; n=1414**	719 (50.8%)
**Maintenance OCS mg; n=718**	10 (8–15)
**ICS dose (BDP equivalent-µg); n=1345**	2000 (1600–2000)
**Biologic therapy; n=1421**	1421 (100.0%)
**Biologic therapy name; n=1159**	
Mepolizumab	606 (52.3%)
Benralizumab	398 (34.3%)
Omalizumab	148 (12.8%)
Dupilumab	7 (0.6%)

Demographics of study cohort at baseline. Mean±sd, median (interquartile range) and counts (%) as appropriate. Medication as taken at baseline except for biologics that are listed as started after baseline assessment. BMI: body mass index; FEV_1_: forced expiratory volume in 1 s; FVC: forced vital capacity; ACQ: asthma control questionnaire; ED: emergency department; *F*_ENO_: exhaled nitric oxide fraction; OCS: oral corticosteroid; ICS: inhaled corticosteroid; BDP: beclomethasone dipropionate equivalent. ^#^: pack-year history unavailable for 20 ex-smokers.

The numbers of UKSAR patients ineligible by the themes of Age, Weight and No Substance Abuse were very small and (proportions) below that required for desired statistical power – these were not further analysed (table 1).

Less than half of patients in UKSAR had bronchodilator reversibility (BDR) and/or bronchial challenge testing at baseline assessment, and of these patients the majority did not show Confirmatory Diagnostic Lung Function RCT eligibility on those lung function studies (41% eligible, table 1, supplementary table S4). However, the majority of patients at baseline assessment had lung function that met the Impaired Lung Function criteria for RCT inclusion (72% eligible, supplementary table S5). The majority of UKSAR patients at baseline also met the Uncontrolled Asthma Symptoms at baseline eligibility criteria (85% eligible, supplementary table S6). Only 8% of patients in this cohort would have been ineligible by preventer inhaler Medication Adherence (92% eligible, supplementary table S7). The majority of patients were eligible by the criteria for RCT studies of No Significant Smoking History (74%, supplementary table S8) and No Comorbid Pulmonary/Other Eosinophilic Disease (73%, supplementary table S9).

Considering all the eligibility criteria together, only a minority of patients were fully eligible by all criteria (15.2% fully eligible).

### Primary outcome: noninferiority for composite response

**FIGURE 1:**
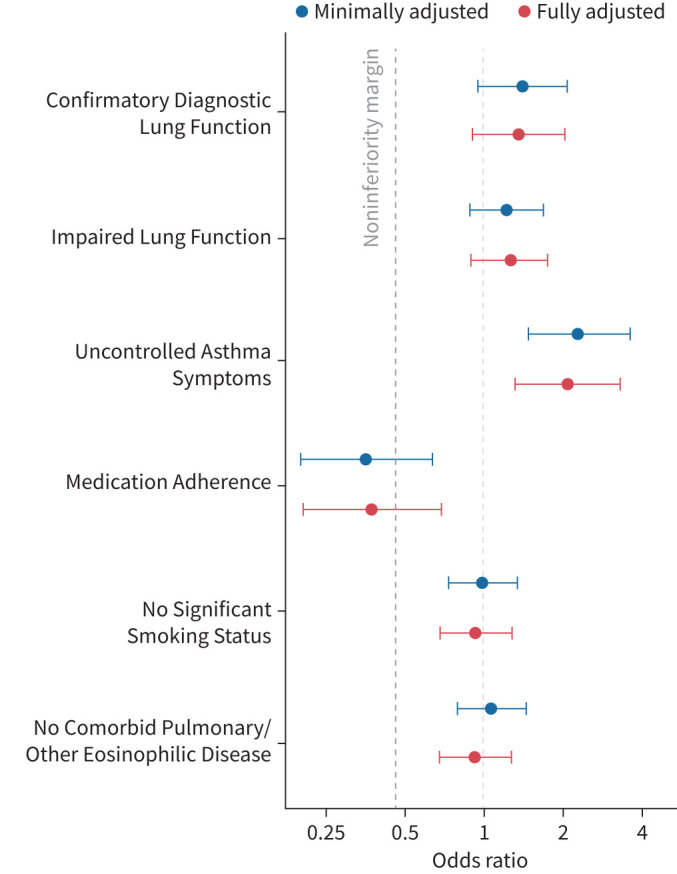
Forest plot of odds ratio for composite response in ineligible patients by each eligibility criteria theme as compared to eligible patients. Odds ratios (ORs) with 95% confidence intervals (CIs) for composite response in ineligible patients *versus* eligible patients for each eligibility criteria theme. The primary outcome of noninferiority is established if the 95% CI lower limit of the OR, comparing the probability of biologic response in the ineligible *versus* eligible group, is >0.46. Minimally adjusted models included adjustment for hospital site. Fully adjusted models additionally included age (5-year categories), sex and pre-biologic blood eosinophil counts. Noninferiority margin shown as dashed line at x-axis intercept 0.46.

Whether UKSAR patients ineligible by each representative inclusion/exclusion criteria had noninferior responses to biologics at first annual review follow-up, compared to eligible patients, was analysed in terms of a composite response of a 50% or more reduction in mOCS and/or in OCS-requiring exacerbations (table 3, figure 1). For all of the eligibility themes except Medication Adherence, there was evidence of noninferiority for achieving a successful composite biologic response at follow-up review. For Medication Adherence the 95% CI for odds ratio for response in ineligible *versus* eligible patients crossed the noninferiority bound – as such there was no evidence of noninferiority for composite response. These findings were consistent between minimal-adjusted and fully adjusted models.

**TABLE 3:** Odds ratios (OR) for composite response in ineligible patients *versus* eligible patients for each eligibility criteria theme

Criteria	Ineligible		Eligible		Minimal adjusted OR (95% CI)	Fully adjusted OR (95% CI)
	Total number	Composite responders (%)	Total number	Composite responders (%)		
**Confirmatory Diagnostic Lung Function**	332	247 (74.4%)	233	161 (69.1%)	1.40 (0.94–2.08)	1.36 (0.90–2.04)
**Impaired Lung Function**	316	247 (78.2%)	809	600 (74.2%)	1.21 (0.88–1.67)	1.25 (0.90–1.73)
**Uncontrolled Asthma Symptoms**	184	156 (84.8%)	1039	773 (74.4%)	2.30 (1.47–3.60)	2.09 (1.31–3.32)
**Medication Adherence**	72	47 (65.3%)	876	663 (75.7%)	0.35 (0.20–0.63)	0.37 (0.20–0.68)
**No Significant Smoking History**	335	255 (76.1%)	979	742 (75.8%)	0.99 (0.73–1.34)	0.93 (0.67–1.27)
**No Comorbid Pulmonary/Other Eosinophilic Disease**	363	279 (76.9%)	996	749 (75.2%)	1.07 (0.80–1.44)	0.92 (0.68–1.25)
**All Criteria (Fully Eligible)**	324	233 (71.9%)	57	37 (64.9%)	1.49 (0.79–2.80)	1.29 (0.66–2.49)

Minimally adjusted models included adjustment for hospital site. Fully adjusted models additionally included age (5-year categories), sex and pre-biologic blood eosinophil counts.

### Secondary outcomes and domain-specific responses

Whilst inferiority (with >15% difference in response rate) was not proven with RCT ineligibility for any of the assessed criteria themes, the composite response rate was significantly lower among those who were ineligible by RCT criteria of Medication Adherence compared to those eligible (fully adjusted OR (95% CI) 0.37 (0.20–0.68)). Conversely, a superior composite response was evident in patients ineligible by the Uncontrolled Asthma Symptoms criteria (fully-adjusted OR 2.09 (1.31–3.32) for achieving composite response in ineligible patients).

Compared to fully eligible patients, those ineligible by one or more criteria had a noninferior composite response to biologics (fully-adjusted OR 1.29 (0.66–2.49)).

**FIGURE 2:**
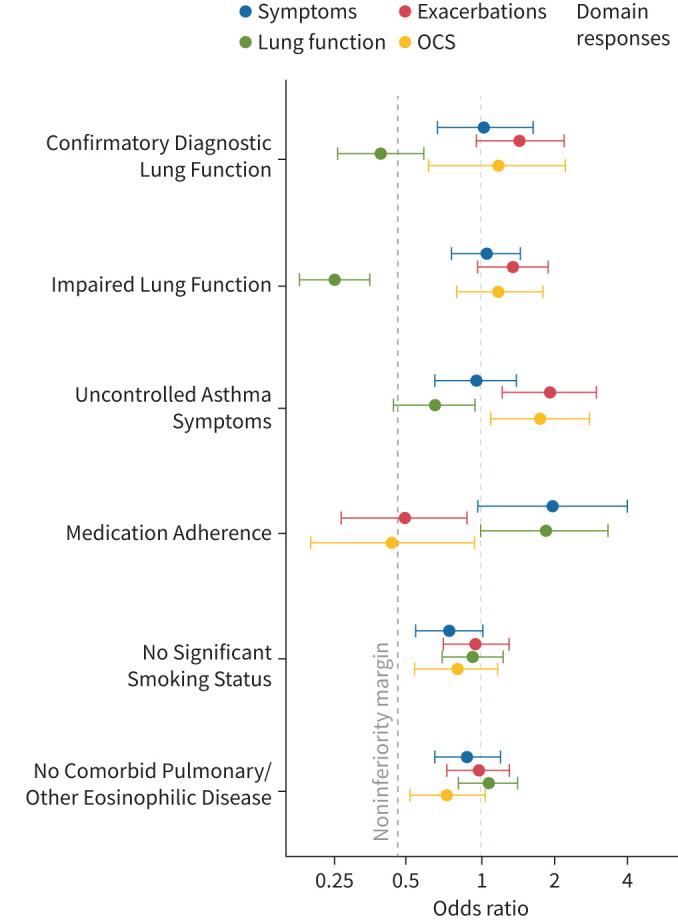
Forest plot of odds ratio for domain-specific responses in ineligible patients by each eligibility criteria theme as compared to eligible patients. Odds ratios (ORs) with 95% confidence intervals (CIs) for domain-specific responses in ineligible patients *versus* eligible patients for each eligibility criteria theme. Noninferiority is established if the 95% CI lower limit of the OR, comparing the probability of biologic response in the ineligible *versus* eligible group, is >0.46. Fully adjusted models include adjustment for hospital site, age (5-year categories), sex and pre-biologic blood eosinophil counts. Noninferiority margin shown as dashed line at x-axis intercept 0.46. Domains: Symptoms, Asthma Control Questionnaire (ACQ) Improvement ≥0.5 or Controlled; Exacerbations, Exacerbation Reduction >50% or No Exacerbations; Lung Function, Forced Expiratory Volume in 1 s increase >100 ml; Oral Corticosteroid (OCS), Maintenance OCS (mOCS) Dose Decrease ≥50% or No mOCS.

We next analysed whether RCT eligibility affected response to biologics across individual domains (daily symptom improvement, exacerbation reduction, lung function improvement and reduced mOCS requirement) (table 4, figure 2).

**TABLE 4:** Odds ratios for domain specific responses in ineligible patients *versus* eligible patients for each eligibility criteria theme

Criteria	Response domain	Ineligible		Eligible		Minimal adjusted OR (95% CI)	Fully adjusted OR (95% CI)
		Total number	Responders (%)	Total number	Responders (%)		
**Confirmatory Diagnostic Lung Function**	ACQ Improvement ≥0.5 or Controlled	278	207 (74.5%)	189	137 (72.5%)	1.09 (0.71–1.68)	1.04 (0.66–1.64)
	Exacerbation Reduction >50% or No Exacerbations	334	247 (74.0%)	234	161 (68.8%)	1.46 (0.98–2.16)	1.44 (0.95–2.16)
	FEV_1_ increase >100 mL	275	117 (42.5%)	199	134 (67.3%)	0.40 (0.27–0.60)	0.39 (0.26–0.59)
	mOCS Dose Decrease ≥50% or No mOCS	132	67 (50.8%)	99	46 (46.5%)	1.27 (0.71–2.27)	1.17 (0.62–2.22)
**Impaired Lung Function**	ACQ Improvement ≥0.5 or Controlled	259	184 (71.0%)	672	464 (69.0%)	1.05 (0.76–1.44)	1.05 (0.75–1.46)
	Exacerbation Reduction >50% or No Exacerbations	317	243 (76.7%)	812	581 (71.6%)	1.31 (0.96–1.79)	1.36 (0.98–1.87)
	FEV_1_ increase >100 mL	279	71 (25.4%)	700	389 (55.6%)	0.26 (0.19–0.35)	0.25 (0.18–0.35)
	mOCS Dose Decrease ≥50% or No mOCS	166	99 (59.6%)	428	236 (55.1%)	1.16 (0.78–1.71)	1.20 (0.81–1.79)
**Uncontrolled Asthma Symptoms**	ACQ Improvement ≥0.5 or Controlled	159	108 (67.9%)	927	642 (69.3%)	1.04 (0.72–1.51)	0.96 (0.65–1.40)
	Exacerbation Reduction >50% or No Exacerbations	184	153 (83.2%)	1043	755 (72.4%)	2.09 (1.37–3.18)	1.92 (1.23–2.97)
	FEV_1_ increase >100 mL	154	62 (40.3%)	872	412 (47.2%)	0.71 (0.50–1.02)	0.65 (0.44–0.94)
	mOCS Dose Decrease ≥50% or No mOCS	113	71 (62.8%)	531	288 (54.2%)	1.66 (1.05–2.62)	1.74 (1.09–2.80)
**Medication Adherence**	ACQ Improvement ≥0.5 or Controlled	58	46 (79.3%)	715	493 (69.0%)	1.70 (0.85–3.39)	1.97 (0.97–3.97)
	Exacerbation Reduction >50% or No Exacerbations	73	48 (65.8%)	877	654 (74.6%)	0.45 (0.26–0.81)	0.49 (0.27–0.88)
	FEV_1_ increase >100 mL	58	33 (56.9%)	726	325 (44.8%)	1.68 (0.94–3.02)	1.82 (1.01–3.28)
	mOCS Dose Decrease ≥50% or No mOCS	39	21 (53.8%)	448	241 (53.8%)	0.45 (0.21–0.96)	0.44 (0.20–0.94)
**No Significant Smoking History**	ACQ Improvement ≥0.5 or Controlled	280	184 (65.7%)	773	543 (70.2%)	0.77 (0.57–1.04)	0.74 (0.54–1.02)
	Exacerbation Reduction >50% or No Exacerbations	338	251 (74.3%)	980	723 (73.8%)	1.03 (0.77–1.39)	0.96 (0.70–1.30)
	FEV_1_ increase >100 mL	282	129 (45.7%)	822	390 (47.4%)	0.93 (0.70–1.23)	0.92 (0.69–1.23)
	mOCS Dose Decrease ≥50% or No mOCS	166	89 (53.6%)	528	297 (56.3%)	0.87 (0.60–1.27)	0.80 (0.54–1.18)
**No Comorbid Pulmonary/Other Eosinophilic Disease**	ACQ Improvement ≥0.5 or Controlled	296	199 (67.2%)	790	551 (69.7%)	0.93 (0.69–1.25)	0.88 (0.64–1.19)
	Exacerbation Reduction >50% or No Exacerbations	364	271 (74.5%)	999	735 (73.6%)	1.10 (0.83–1.47)	0.97 (0.72–1.31)
	FEV_1_ increase >100 mL	312	147 (47.1%)	830	389 (46.9%)	1.08 (0.83–1.42)	1.07 (0.81–1.42)
	mOCS Dose Decrease ≥50% or No mOCS	230	124 (53.9%)	486	273 (56.2%)	0.77 (0.55–1.09)	0.73 (0.52–1.04)
**All Criteria (Fully Eligible)**	ACQ Improvement ≥0.5 or Controlled	300	227 (75.7%)	51	30 (58.8%)	2.25 (1.18–4.30)	2.01 (1.01–4.00)
	Exacerbation Reduction >50% or No Exacerbations	325	235 (72.3%)	57	38 (66.7%)	1.52 (0.80–2.89)	1.24 (0.62–2.46)
	FEV_1_ increase >100 mL	269	119 (44.2%)	51	36 (70.6%)	0.34 (0.18–0.67)	0.30 (0.15–0.60)
	mOCS Dose Decrease ≥50% or No mOCS	132	69 (52.3%)	20	5 (25.0%)	3.29 (1.03–10.48)	2.71 (0.78–9.39)

Minimally adjusted models included adjustment for hospital site. Fully adjusted models additionally included age (5-year categories), sex and pre-biologic blood eosinophil counts. ACQ: asthma control questionnaire; FEV_1_: forced expiratory volume in 1 s; mOCS: maintenance oral corticosteroid.

Inferiority of response in the lung function improvement (FEV_1_ increase >100 mL) domain was evident in those patients ineligible by the Impaired Lung Function criteria for RCT inclusion. Noninferiority in lung function domain responses could also not be demonstrated in patients ineligible by the criteria of Confirmatory Diagnostic Lung Function or Uncontrolled Asthma Symptoms (fully adjusted models). Additionally, in patients ineligible by Medication Adherence, noninferiority of response could not be demonstrated in the domains of exacerbation reduction and reduced mOCS requirement.

Superior responses were seen in the domains of exacerbation reduction and reduced mOCS requirement in patients ineligible through not having Uncontrolled Asthma Symptoms at baseline. A superior response was also seen in the lung function improvement domain in patients ineligible through reduced Medication Adherence.

Those patients ineligible by one or more criteria, compared to fully eligible patients, had a superior response to biologics in the domain of symptom improvement but significantly lower response rate in the domain of lung function improvement.

### Sensitivity analyses

There were no significant differences in composite response or domain-specific responses (supplementary tables S10, S11 and S12) comparing patients identified by these diagnostic definitions as eligible and ineligible through comorbid COPD or ABPA, though the number of patients identified as meeting the diagnostic definition of ABPA was very small.

## Discussion

Phase 3 RCTs have previously shown the efficacy of biologics in the management of “ideal” patients with severe asthma, and phase 4 observational studies and real-world registry research the effectiveness of biologics in heterogeneous global populations of patients with severe asthma, many of whom would not have met the inclusion/exclusion criteria of the preceding phase 3 RCTs. In this research we have extended those findings to study whether specific inclusion/exclusion criteria used in the RCTs might distinguish patients who do not achieve adequate clinical responses to biologics. We have found none of the criteria identify patient groups who have significantly impaired clinical outcomes on biologic therapies, as determined using noninferiority testing, although significant noninferiority could not be demonstrated for patients ineligible through inhaled medication nonadherence.

We chose noninferiority testing and a composite outcome of reduction in exacerbations and/or maintenance OCS for the primary outcome given the aim to establish whether ineligibility by any of the examined criteria was associated with a sufficiently reduced likelihood for a biologic response to suggest use of biologics in such patients should potentially be discouraged. A consequence of that aim was the notably low noninferiority bound of 0.46 for the odds ratio in ineligible *versus* eligible patients. The composite response rate for fully eligible patients in this study was 71.9%, lower than the *a priori* estimate of 80%, and which would have resulted in a noninferiority bound of 0.52 but no change in the overall results of the study. The noninferiority bound is also affected by the size of the noninferiority margin, and a limitation of noninferiority testing is the lack of defined standards for determining noninferiority margins [28]. Importantly, statistically noninferior responses in ineligible patients does not preclude superior responses in eligible patients, as noninferiority studies by design only assess for differences greater than the threshold set by the noninferiority margin.

We therefore also conducted superiority analyses as secondary outcomes to better describe differences in response between eligible and ineligible patients, and found significant associations with biologic response for several eligibility criteria. Few previous studies have examined associations with individual domain responses and where they have, only whether baseline impairment in a domain predicts response in the same domain [29].

Interestingly although only a small proportion of UKSAR patients were identified as ineligible through having controlled asthma symptoms (ACQ-6 <1.5) at baseline, these patients without significant asthma symptoms when being assessed between exacerbations had superior responses to biologics in terms of reduced exacerbations and reduced requirement for mOCS. Symptoms of breathlessness between exacerbations may relate more to treatable traits such as fixed airflow obstruction, breathing pattern disorders, deconditioning and obesity rather than uncontrolled T2 airways inflammation [30–32], and these comorbidities could potentially also be associated with reduced biologic response [33]. The International Severe Asthma Registry and a recent meta-analysis of determinants of remission have similarly shown patients with better pre-biologic asthma symptom control to have greater likelihood of achieving clinical remission on biologics [34, 35].

Many of the patients in UKSAR did not exhibit either BDR or bronchial hyperreactivity at baseline, yet they responded similarly to biologics. The German Severe Asthma Registry has similarly reported a high proportion of real-world patients with severe asthma to have negative BDR testing [36]. Consistent with our results, Mümmler and colleagues [37] did not find presence or absence of BDR at baseline to significantly affect response to severe asthma biologics in terms of exacerbation reduction or ability to stop maintenance OCS. Chronic airway remodelling in patients may explain the absence of BDR in some; however, it is important to appreciate that BDR in individual patients exhibits temporal variation [38]. To allow for this, most clinical trials allow for repeated BDR testing over the screening period, with positive BDR on one occasion sufficient for trial eligibility. UKSAR only records the results of a single BDR test at baseline. However, it is important to consider that these patients did undergo systematic assessment to exclude other conditions and may have had other or previous diagnostic evidence for asthma [12]. Ultimately our results are supportive of the multidisciplinary systematic assessment approach to diagnosing severe asthma rather than focussing on single diagnostic tests.

Lack of diagnostic lung function studies and of impaired FEV_1_ at baseline was associated with reduced likelihood of lung function improvement on biologics. Improving lung function may simply be unrealistic in those patients with good lung function at baseline – a ceiling effect – though the International Severe Asthma Registry (ISAR) has shown that a minority of patients with baseline FEV_1_ ≥80% predicted can achieve increases in FEV_1_ of 100 mLor more post-biologic with some showing increases of 500 mLor more [29]. In contrast to our results, Mümmler and colleagues [37] did not find baseline BDR to be associated with significant differences in FEV_1_ increase on biologic therapy.

Clinically COPD can often be difficult to differentiate from severe asthma, and there is increasing interest in the benefits of biologics in COPD. However, in many clinical trials of COPD outcomes for anti-interleukin (IL)-5(R) biologics have not been as impressive as seen in equivalent trials in severe asthma [39, 40]. Nevertheless, Shim and colleagues [41] found no significant clinical differences in biologic response in patients with asthma meeting, compared to not-meeting, criteria to be defined as having asthma–COPD overlap in the PRISM cohort. Consistent with their findings, in UKSAR, patients with a smoking history had noninferior response to biologics. Differences in outcomes between biologics clinical trials in asthma and COPD may simply reflect other differences in trial design.

### Limitations

Whilst most of the relevant variables for these analyses and some comorbidities are captured in UKSAR using specific numeric/categorical fields, other comorbidities are only captured through searches of free-text fields [12]. It is possible that some patients with these comorbidities were not identified as such in this analysis, resulting in some potential misclassification. We therefore undertook sensitivity analyses in which alternate definitions of specific comorbidities by which patients might be ineligible were considered though these did not alter our findings.

The extent to which surrogates of RCT inclusion/exclusion criteria can be operationally defined in UKSAR also varies across different criteria. In particular measures of long-term medication prescription refill in UKSAR do not necessarily mirror medication adherence during a short run-in period during a clinical trial. Indeed, improvements in medication adherence during trials may contribute to significant placebo effects seen in many asthma trials [42].

The majority of patients included in this study were on anti-IL-5(R) biologics. Potentially responses to other asthma biologics may be more influenced by the criteria studied here; however, we were unable to address that question within the UKSAR population. The relative predominance of mepolizumab and benralizumab as first-line in UKSAR may reflect the additional complexity of intravenous administration with reslizumab and current National Institute for Health and Care Excellence (NICE) criteria for dupilumab approval that requires it to effectively be a second-line biologic in eosinophilic patients. Notably the influence of medication adherence on biologic response does differ between specific biologics [43, 44]. Our study was not powered to compare between biologics the effect of eligibility criteria on response, and as the number of available biologics and size of severe asthma registries grow, these analyses will need repeating for individual biologics.

In this paper we chose a composite response for our primary outcome based on the response requirement for continuing UK biologic prescription reimbursement as determined by NICE throughout most of the study period, which in turn is based upon a health economic analysis of the benefits of reducing systemic corticosteroid exposure [45]. As per UK reimbursement criteria, all patients received biologics to reduce systemic corticosteroid exposure and exacerbations. In some countries biologics are available for patients with neither frequent exacerbations nor systemic corticosteroid exposure [10], and future research is needed to assess factors determining biologic response in these patients. Whilst we do here report other quantitative response domains such as symptom score, it is important to note that patients view response to biologics in a more complex manner in terms of reduced burden of both disease and treatment, and in particular in relation to their individual activities [46]. As such, patient views on positive biologic response do not always match the quantitative definitions used in studies.

### Conclusion

In this multicentre analysis, ineligibility by typical RCT inclusion/exclusion themes was associated with noninferiority of response to biologics with the exception of medication adherence where noninferiority could not be demonstrated. Whilst strict inclusion/exclusion criteria are needed for RCTs leading to drug licensing, our results show that asthma biologics are effective in a broad range of patients, many of whom would not have met clinical trial criteria. Multidisciplinary systematic assessment should remain the gold standard in clinical services for identifying patients with severe asthma who might benefit from biologic therapy, and arguably in future clinical trials.

## Supplementary material

**Please note:** supplementary material is not edited by the Editorial Office, and is uploaded as it has been supplied by the author. Supplementary material 00565-2025.SUPPLEMENT
