# Supplementary material for: Real-world effectiveness of biologic therapies in severe asthma patients ineligible for phase 3 randomised controlled trials of biologics: an analysis from the UK Severe Asthma Registry
Source: ERJ Open Res. 2026 Jan 19;12(1):00565-2025. doi: 10.1183/23120541.00565-2025 (PMC12813675; doi:10.1183/23120541.00565-2025)
Supplement: Supplementary file 1 [file 00565-2025.SUPPLEMENT.pdf]

Supplementary Table S1

Variable		Missing (%)	Complete (%)
Baseline			
	Age At First Assessment (Years)	0/1421 (0.0%)	1421/1421 (100.0%)
	Age of Onset (Years)	95/1421 (6.7%)	1326/1421 (93.3%)
	Gender	0/1421 (0.0%)	1421/1421 (100.0%)
	Ethnicity	17/1421 (1.2%)	1404/1421 (98.8%)
	BMI (kg/m2)	12/1421 (0.8%)	1409/1421 (99.2%)
	Smoking Status	28/1421 (2.0%)	1393/1421 (98.0%)
	Atopic Disease	20/1421 (1.4%)	1401/1421 (98.6%)
	FEV1 (L)	239/1421 (16.8%)	1182/1421 (83.2%)
	FEV1 (% Predicted)	247/1421 (17.4%)	1174/1421 (82.6%)
	FVC (L)	254/1421 (17.9%)	1167/1421 (82.1%)
	FVC (% Predicted)	298/1421 (21.0%)	1123/1421 (79.0%)
	FEV1/FVC	258/1421 (18.2%)	1163/1421 (81.8%)
	ACQ6 Score	148/1421 (10.4%)	1273/1421 (89.6%)
	Uncontrolled Asthma (ACQ6≥1.5)	148/1421 (10.4%)	1273/1421 (89.6%)
	Exacerbations (Last Year)	20/1421 (1.4%)	1401/1421 (98.6%)
	Any ED Attendance (Last Year)	26/1421 (1.8%)	1395/1421 (98.2%)
	Any Hospital Admissions (Last Year)	22/1421 (1.5%)	1399/1421 (98.5%)
	Invasive Ventilations (Ever)	29/1421 (2.0%)	1392/1421 (98.0%)
	Blood Eosinophil Count ($x10^9$/L)	19/1421 (1.3%)	1402/1421 (98.7%)
	Highest Blood Eosinophil Count ($x10^9$/L)	26/1421 (1.8%)	1395/1421 (98.2%)
	FeNO (ppb)	284/1421 (20.0%)	1137/1421 (80.0%)
	IgE (IU/mL)	70/1421 (4.9%)	1351/1421 (95.1%)
	Maintenance OCS	7/1421 (0.5%)	1414/1421 (99.5%)
	Maintenance OCS (mg)	1/719 (0.1%)	718/719 (99.9%)
	Biologic Therapy	0/1421 (0.0%)	1421/1421 (100.0%)
	Biological Therapy Name	59/1218 (4.8%)	1159/1218 (95.2%)
Follow-up			
	Exacerbations (Last Year)	39/1421 (2.7%)	1382/1421 (97.3%)
	Exacerbations (Last Year)	39/1421 (2.7%)	1382/1421 (97.3%)
	Any ED Attendance (Last Year)	57/1421 (4.0%)	1364/1421 (96.0%)
	Any Hospital Admissions (Last Year)	56/1421 (3.9%)	1365/1421 (96.1%)
	Blood Eosinophil Count ($x10^9$L)	455/1421 (32.0%)	966/1421 (68.0%)
	FeNO (ppb)	545/1421 (38.4%)	876/1421 (61.6%)
	ACQ6 Score	241/1421 (17.0%)	1180/1421 (83.0%)
	Uncontrolled Asthma (ACQ6≥1.5)	241/1421 (17.0%)	1180/1421 (83.0%)
	Maintenance OCS	7/1421 (0.5%)	1414/1421 (99.5%)
	Maintenance OCS (mg)	4/605 (0.7%)	601/605 (99.3%)
	Difference ACQ6 Score	335/1421 (23.6%)	1086/1421 (76.4%)
	Difference EuroQoL Utility	890/1421 (62.6%)	531/1421 (37.4%)
	% Difference Exacerbations (Last Year)	58/1313 (4.4%)	1255/1313 (95.6%)

	% Difference Blood Eosinophil Count	466/1377 (33.8%)	911/1377 (66.2%)
	% Difference FeNO	599/1421 (42.2%)	822/1421 (57.8%)
	Difference Clinic FEV1 (L)	279/1421 (19.6%)	1142/1421 (80.4%)
	ACQ Improvement≥0.5 or Controlled	335/1421 (23.6%)	1086/1421 (76.4%)
	Exacerbation Reduction>50% or No Exacerbations	58/1421 (4.1%)	1363/1421 (95.9%)
	Unscheduled Care Reduction≥50% or No Unscheduled	78/1421 (5.5%)	1343/1421 (94.5%)
	FEV1 increase>100ml	279/1421 (19.6%)	1142/1421 (80.4%)
	OCS Dose Decrease≥50% or No OCS	705/1421 (49.6%)	716/1421 (50.4%)
	Composite Response	62/1421 (4.4%)	1359/1421 (95.6%)

Suppl Table S1: Data Completeness and Analysis of Missing Data

Supplementary Table S2

**Free-text search terms for comorbidities identifying patients as ineligible by 'No Substance Abuse' criteria for RCT studies**	"alcohol[a-z\-]*" "illicit" "EtOH" "methadone" "IVDU" "drug use[a-z]*" "heroin" "cocaine" "cannabis" "recreational drug"
**Free-text search terms for comorbidities identifying patients as ineligible by 'No Comorbid Pulmonary / Other Eosinophilic Disease' criteria for RCT studies**	"copd" "emphysema" "acos" "abpa" "aspergillosis" "abpm" "egpa" "churg[a-z\-]+" "polyangi[a-z]+" "bronchiectasis" "pulmonary fibrosis" "ipf" "interstitial lung disease" "ild" "uip" "nsip" "cryptogenic fibrosing alveolitis" "cystic fibrosis" "cf" "obesity hypoventilation" "ohs" "primary ciliary dyskinesia" "pcd" "antitrypsin deficiency" "lung cancer" "non-small cell cancer" "small cell cancer" "hypereosinophilic syndrome" "carcinoid" "bronchogenic carcinoma" "bronchogenic malignancy"

Suppl Table S2: Search terms in UKSAR utilised to identify eligible / ineligible patients for specific themes

Supplementary Table S3

Patients Follow-Up Characteristics (N= number of patients)	Entire Cohort (N=1421)
**Follow-up Time (Days); N=1421**	404.0 (364.0,495.0)
**BMI (kg/m2); N=1116**	31.0 (7.3)
**Exacerbations (Last Year); N=1382**	1 (0,3)
**Any ED Attendance (Last Year); N=1364**	190 (13.9%)
**Blood Eosinophil Count (x10^9L); N=966**	0.04 (0.00,0.13)
**FeNO (ppb); N=876**	37 (20,70)
**ACQ6 Score; N=1180**	1.8 (0.7,3.0)
**EuroQoL Utility; N=592**	0.80 (0.54,0.95)
**Maintenance OCS; N=1414**	605 (42.8%)
**Maintenance OCS (mg); N=601**	6 (5,10)
**Difference ACQ6 Score; N=1086**	-0.8 (-1.8,0.0)
**% Difference Exacerbations (Last Year); N=1255**	-80.0 (-100.0,-42.9)
**% Difference Blood Eosinophil Count; N=911**	-87.5 (-100.0,-35.6)
**% Difference FeNO (ppb); N=822**	-11.9 (-44.3,37.5)
**Maintenance OCS Change; N=714**	
Discontinue	191 (26.8%)
Decrease Dose	326 (45.7%)
Maintain Dose	143 (20.0%)
Increase Dose	54 (7.6%)
**ACQ Improvement ≥0.5 or Controlled; N=1086**	750 (69.1%)
**Exacerbation Reduction >50% or No Exacerbations; N=1363**	1,006 (73.8%)
**FEV1 increase >100ml; N=1142**	536 (46.9%)
**Maintenance OCS Dose Decrease ≥50% or No mOCS; N=716**	397 (55.4%)
**Composite Response; N=1359**	1,028 (75.6%)

Suppl Table S3: Demographics of severe asthma patient cohort included in this analysis at follow-up. Means (standard deviation [SD]), medians (interquartile ranges [IQR]) and counts (percentages) as appropriate. ACQ; asthma control questionnaire. ED; emergency department. mOCS; maintenance oral corticosteroid.

Supplementary Table S4

**Confirmatory Diagnostic Lung Function**	**Entire Cohort**	**No (ineligible)**	**Yes (eligible)**	**P-value**
**Number of Patients; N=579**	**579**	**341**	**238**	
Age At First Assessment (Years); N=579	50.9 (15.5)	51.6 (14.8)	49.8 (16.5)	0.171
Age of Onset (Years); N=546	26.9 (21.0)	27.5 (21.0)	26.0 (20.9)	0.433
Gender; N=579				0.495
Female	348 (60.1%)	201 (58.9%)	147 (61.8%)	
Ethnicity; N=573				0.965
Caucasian	484 (84.5%)	284 (84.5%)	200 (84.4%)	
Non-Caucasian	89 (15.5%)	52 (15.5%)	37 (15.6%)	
BMI (kg/m2); N=577	31.2 (8.1)	31.4 (8.5)	30.9 (7.4)	0.485
Exacerbations (Last Year); N=574	5 (3,8)	5 (3,8)	5 (4,8)	0.682
Blood Eosinophil Count ($x10^9$/L); N=574	0.40 (0.20,0.66)	0.32 (0.14,0.60)	0.40 (0.23,0.70)	0.017
Highest Blood Eosinophil Count ($x10^9$/L); N=567	0.70 (0.48,1.16)	0.72 (0.50,1.20)	0.70 (0.45,1.07)	0.113
FeNO (ppb); N=501	44 (25,74)	38 (24,69)	53 (28,84)	0.004
Maintenance OCS; N=576	233 (40.5%)	134 (39.6%)	99 (41.6%)	0.638
Inc/Exc Criteria: Diagnostic RFTs; N=579	238 (41.1%)	0 (0.0%)	238 (100.0%)	<0.001
Inc/Exc Criteria: FEV1<80%; N=575	417 (72.5%)	212 (62.5%)	205 (86.9%)	<0.001
Inc/Exc Criteria: ACQ6≥1.5; N=532	459 (86.3%)	266 (84.2%)	193 (89.4%)	0.088
Inc/Exc Criteria: MPR≥70%; N=431	403 (93.5%)	251 (94.7%)	152 (91.6%)	0.197
Inc/Exc Criteria: No Significant Smoking History; N=559	391 (69.9%)	233 (70.4%)	158 (69.3%)	0.781
Inc/Exc Criteria: No comorbid lung or eosinophilic disease; N=579	444 (76.7%)	254 (74.5%)	190 (79.8%)	0.134
Inc/Exc Criteria: Fully Eligible; N=389	59 (15.2%)	0 (0.0%)	59 (40.4%)	<0.001

Suppl Table S4: Characteristics at baseline of patients eligible / ineligible by the criteria of Confirmatory Diagnostic Lung Function

Supplementary Table S5

**Impaired Lung Function**	**Entire Cohort**	**No (ineligible)**	**Yes (eligible)**	**P-value**
**Number of Patients; N=1174**	**1,174**	**326**	**848**	
Age At First Assessment (Years); N=1174	51.4 (14.4)	49.9 (14.9)	52.0 (14.2)	0.021
Age of Onset (Years); N=1101	26.1 (20.0)	26.8 (19.5)	25.8 (20.2)	0.449
Gender; N=1174				0.010
Female	708 (60.3%)	216 (66.3%)	492 (58.0%)	
Ethnicity; N=1168				0.010
Caucasian	980 (83.9%)	288 (88.3%)	692 (82.2%)	
Non-Caucasian	188 (16.1%)	38 (11.7%)	150 (17.8%)	
BMI (kg/m2); N=1174	30.7 (7.4)	30.8 (7.9)	30.6 (7.1)	0.690
Exacerbations (Last Year); N=1158	5 (3,8)	5 (3,7)	5 (3,8)	0.673
Blood Eosinophil Count (x10^9/L); N=1164	0.37 (0.20,0.60)	0.30 (0.15,0.59)	0.40 (0.20,0.60)	0.005
Highest Blood Eosinophil Count (x10^9/L); N=1155	0.70 (0.47,1.10)	0.71 (0.42,1.17)	0.70 (0.48,1.10)	0.688
FeNO (ppb); N=963	44 (24,77)	44 (24,81)	44 (25,77)	0.869
Maintenance OCS; N=1167	597 (51.2%)	167 (51.4%)	430 (51.1%)	0.923
Inc/Exc Criteria: Diagnostic RFTs; N=575	236 (41.0%)	31 (19.6%)	205 (49.2%)	<0.001
Inc/Exc Criteria: FEV1<80%; N=1174	848 (72.2%)	0 (0.0%)	848 (100.0%)	<0.001
Inc/Exc Criteria: ACQ6≥1.5; N=1071	914 (85.3%)	232 (77.3%)	682 (88.5%)	<0.001
Inc/Exc Criteria: MPR≥70%; N=861	785 (91.2%)	234 (93.6%)	551 (90.2%)	0.108
Inc/Exc Criteria: No Significant Smoking History; N=1135	836 (73.7%)	248 (80.0%)	588 (71.3%)	0.003
Inc/Exc Criteria: No comorbid lung or eosinophilic disease; N=1174	854 (72.7%)	239 (73.3%)	615 (72.5%)	0.786
Inc/Exc Criteria: Fully Eligible; N=389	59 (15.2%)	0 (0.0%)	59 (21.1%)	<0.001

Suppl Table S5: Characteristics at baseline of patients eligible / ineligible by the criteria of Impaired Lung Function

Supplementary Table S6

**Uncontrolled Asthma Symptoms**	**Entire Cohort**	**No (ineligible)**	**Yes (eligible)**	**P-value**
**Number of Patients; N=1273**	**1,273**	**192**	**1,081**	
Age At First Assessment (Years); N=1273	51.7 (14.7)	57.1 (13.6)	50.7 (14.7)	<0.001
Age of Onset (Years); N=1190	26.4 (20.2)	37.2 (19.9)	24.4 (19.6)	<0.001
Gender; N=1273				<0.001
Female	758 (59.5%)	86 (44.8%)	672 (62.2%)	
Ethnicity; N=1256				0.021
Caucasian	1,058 (84.2%)	169 (89.9%)	889 (83.2%)	
Non-Caucasian	198 (15.8%)	19 (10.1%)	179 (16.8%)	
BMI (kg/m2); N=1264	30.6 (7.2)	28.0 (6.0)	31.0 (7.3)	<0.001
Exacerbations (Last Year); N=1262	5 (3,8)	4 (1,6)	5 (3,8)	<0.001
Blood Eosinophil Count (x10^9/L); N=1257	0.37 (0.20,0.60)	0.39 (0.20,0.63)	0.37 (0.20,0.60)	0.632
Highest Blood Eosinophil Count (x10^9/L); N=1254	0.70 (0.43,1.10)	0.90 (0.50,1.40)	0.70 (0.40,1.03)	<0.001
FeNO (ppb); N=1029	44 (25,79)	51 (28,91)	44 (24,77)	0.020
Maintenance OCS; N=1267	647 (51.1%)	113 (59.5%)	534 (49.6%)	0.012
Inc/Exc Criteria: Diagnostic RFTs; N=532	216 (40.6%)	23 (31.5%)	193 (42.0%)	0.088
Inc/Exc Criteria: FEV1<80%; N=1071	771 (72.0%)	89 (56.7%)	682 (74.6%)	<0.001
Inc/Exc Criteria: ACQ6≥1.5; N=1273	1,081 (84.9%)	0 (0.0%)	1,081 (100.0%)	<0.001
Inc/Exc Criteria: MPR≥70%; N=899	826 (91.9%)	130 (96.3%)	696 (91.1%)	0.042
Inc/Exc Criteria: No Significant Smoking History; N=1232	909 (73.8%)	144 (77.0%)	765 (73.2%)	0.277
Inc/Exc Criteria: No comorbid lung or eosinophilic disease; N=1273	929 (73.0%)	126 (65.6%)	803 (74.3%)	0.013
Inc/Exc Criteria: Fully Eligible; N=389	59 (15.2%)	0 (0.0%)	59 (17.4%)	0.001

Suppl Table S6: Characteristics at baseline of patients eligible / ineligible by the criteria of Uncontrolled Asthma Symptoms

Supplementary Table S7

Medication Adherence	Entire Cohort	No (ineligible)	Yes (eligible)	P-value
**Number of Patients; N=996**	**996**	**81**	**915**	
Age At First Assessment (Years); N=996	50.9 (14.3)	47.9 (15.2)	51.2 (14.2)	0.049
Age of Onset (Years); N=931	25.6 (19.9)	23.2 (20.4)	25.9 (19.8)	0.251
Gender; N=996				0.946
Female	599 (60.1%)	49 (60.5%)	550 (60.1%)	
Ethnicity; N=989				0.007
Caucasian	807 (81.6%)	57 (70.4%)	750 (82.6%)	
Non-Caucasian	182 (18.4%)	24 (29.6%)	158 (17.4%)	
BMI (kg/m2); N=988	30.6 (7.1)	30.0 (8.2)	30.7 (7.0)	0.391
Exacerbations (Last Year); N=981	5 (3,8)	4 (2,7)	5 (3,8)	0.019
Blood Eosinophil Count ($x10^9$/L); N=982	0.37 (0.20,0.60)	0.40 (0.10,0.50)	0.37 (0.20,0.60)	0.161
Highest Blood Eosinophil Count ($x10^9$/L); N=984	0.70 (0.40,1.10)	0.70 (0.46,1.00)	0.70 (0.40,1.10)	0.941
FeNO (ppb); N=779	44 (25,82)	44 (24,88)	44 (25,81)	0.990
Maintenance OCS; N=991	489 (49.3%)	40 (49.4%)	449 (49.3%)	0.994
Inc/Exc Criteria: Diagnostic RFTs; N=431	166 (38.5%)	14 (50.0%)	152 (37.7%)	0.197
Inc/Exc Criteria: FEV1<80%; N=861	611 (71.0%)	60 (78.9%)	551 (70.2%)	0.108
Inc/Exc Criteria: ACQ6≥1.5; N=899	764 (85.0%)	68 (93.2%)	696 (84.3%)	0.042
Inc/Exc Criteria: MPR≥70%; N=996	915 (91.9%)	0 (0.0%)	915 (100.0%)	<0.001
Inc/Exc Criteria: No Significant Smoking History; N=962	709 (73.7%)	59 (74.7%)	650 (73.6%)	0.836
Inc/Exc Criteria: No comorbid lung or eosinophilic disease; N=996	719 (72.2%)	58 (71.6%)	661 (72.2%)	0.903
Inc/Exc Criteria: Fully Eligible; N=389	59 (15.2%)	0 (0.0%)	59 (16.2%)	0.029

Suppl Table S7: Characteristics at baseline of patients eligible / ineligible by the criteria of Medication Adherence

Supplementary Table S8

**No Significant Smoking History**	**Entire Cohort**	**No (ineligible)**	**Yes (eligible)**	**P-value**
**Number of Patients; N=1373**	**1,373**	**357**	**1,016**	
Age At First Assessment (Years); N=1373	51.7 (14.7)	53.4 (13.7)	51.1 (14.9)	0.009
Age of Onset (Years); N=1286	26.0 (20.0)	29.6 (21.4)	24.8 (19.4)	<0.001
Gender; N=1373				<0.001
Female	827 (60.2%)	185 (51.8%)	642 (63.2%)	
Ethnicity; N=1357				0.002
Caucasian	1,145 (84.4%)	315 (89.5%)	830 (82.6%)	
Non-Caucasian	212 (15.6%)	37 (10.5%)	175 (17.4%)	
BMI (kg/m2); N=1364	30.7 (7.4)	31.1 (7.3)	30.6 (7.5)	0.219
Exacerbations (Last Year); N=1356	5 (3,8)	6 (4,8)	5 (3,7)	0.004
Blood Eosinophil Count ($x10^9$/L); N=1355	0.37 (0.20,0.60)	0.34 (0.20,0.56)	0.38 (0.19,0.63)	0.118
Highest Blood Eosinophil Count ($x10^9$/L); N=1347	0.70 (0.42,1.10)	0.70 (0.45,1.00)	0.70 (0.40,1.16)	0.111
FeNO (ppb); N=1109	44 (24,77)	38 (20,69)	47 (26,83)	<0.001
Maintenance OCS; N=1367	697 (51.0%)	169 (47.5%)	528 (52.2%)	0.123
Inc/Exc Criteria: Diagnostic RFTs; N=559	228 (40.8%)	70 (41.7%)	158 (40.4%)	0.781
Inc/Exc Criteria: FEV1<80%; N=1135	825 (72.7%)	237 (79.3%)	588 (70.3%)	0.003
Inc/Exc Criteria: ACQ6≥1.5; N=1232	1,045 (84.8%)	280 (86.7%)	765 (84.2%)	0.277
Inc/Exc Criteria: MPR≥70%; N=962	883 (91.8%)	233 (92.1%)	650 (91.7%)	0.836
Inc/Exc Criteria: No Significant Smoking History; N=1373	1,016 (74.0%)	0 (0.0%)	1,016 (100.0%)	<0.001
Inc/Exc Criteria: No comorbid lung or eosinophilic disease; N=1373	1,000 (72.8%)	236 (66.1%)	764 (75.2%)	<0.001
Inc/Exc Criteria: Fully Eligible; N=389	59 (15.2%)	0 (0.0%)	59 (21.7%)	<0.001

Suppl Table S8: Characteristics at baseline of patients eligible / ineligible by the criteria of No Significant Smoking History

Supplementary Table S9

**No Comorbid Pulmonary / Other Eosinophilic Disease**	**Entire Cohort**	**No (ineligible)**	**Yes (eligible)**	**P-value**
**Number of Patients; N=1421**	**1,421**	**382**	**1,039**	
Age At First Assessment (Years); N=1421	51.6 (14.7)	55.9 (13.0)	50.0 (15.0)	<0.001
Age of Onset (Years); N=1326	26.1 (20.0)	27.1 (20.5)	25.7 (19.9)	0.241
Gender; N=1421				0.001
Female	857 (60.3%)	204 (53.4%)	653 (62.8%)	
Ethnicity; N=1404				0.759
Caucasian	1,186 (84.5%)	322 (85.0%)	864 (84.3%)	
Non-Caucasian	218 (15.5%)	57 (15.0%)	161 (15.7%)	
BMI (kg/m2); N=1409	30.7 (7.4)	28.9 (6.4)	31.3 (7.6)	<0.001
Exacerbations (Last Year); N=1401	5 (3,8)	4 (3,7)	5 (3,8)	<0.001
Blood Eosinophil Count ($x10^9$/L); N=1402	0.38 (0.20,0.60)	0.40 (0.18,0.60)	0.37 (0.20,0.60)	0.952
Highest Blood Eosinophil Count ($x10^9$/L); N=1395	0.70 (0.43,1.10)	0.80 (0.50,1.30)	0.70 (0.40,1.03)	<0.001
FeNO (ppb); N=1137	44 (24,77)	44 (24,81)	44 (24,77)	0.679
Maintenance OCS; N=1414	719 (50.8%)	231 (60.8%)	488 (47.2%)	<0.001
Inc/Exc Criteria: Diagnostic RFTs; N=579	238 (41.1%)	48 (35.6%)	190 (42.8%)	0.134
Inc/Exc Criteria: FEV1<80%; N=1174	848 (72.2%)	233 (72.8%)	615 (72.0%)	0.786
Inc/Exc Criteria: ACQ6≥1.5; N=1273	1,081 (84.9%)	278 (80.8%)	803 (86.4%)	0.013
Inc/Exc Criteria: MPR≥70%; N=996	915 (91.9%)	254 (91.7%)	661 (91.9%)	0.903
Inc/Exc Criteria: No Significant Smoking History; N=1373	1,016 (74.0%)	252 (67.6%)	764 (76.4%)	<0.001
Inc/Exc Criteria: No comorbid lung or eosinophilic disease; N=1421	1,039 (73.1%)	0 (0.0%)	1,039 (100.0%)	<0.001
Inc/Exc Criteria: Fully Eligible; N=389	59 (15.2%)	0 (0.0%)	59 (20.2%)	<0.001

Suppl Table S9: Characteristics at baseline of patients eligible / ineligible by the criteria of No Comorbid Pulmonary / Other Eosinophilic Disease

Supplementary Table S10

Criteria Theme	Operational Definition	Number of Patients Eligible *	Number of Patients Ineligible *
Ineligibility for Suspected comorbid COPD	INELIGIBILITY:<br>Current smoker or ex-smoker with ≥10 pack years smoking history,<br>AND<br>post-bronchodilator FEV1/FVC < 0.7;<br>where post-bronchodilator spirometry unavailable then pre-bronchodilator / random FEV1/FVC < 0.7	1152<br>(87.4%)	166<br>(12.6%)
Ineligibility for Suspected ABPA	INELIGIBILITY:<br>Baseline total IgE > 1000 and positive allergic sensitisation to aspergillus	1308<br>(96.8%)	43<br>(3.2%)

Suppl Table S10: Eligibility criteria for Phase 3 RCTs of biologics in severe asthma, surrogate criteria in UKSAR and numbers of patients eligible/ineligible for eligibility sensitivity analysis

Pre-/post-bronchodilator refers to post- inhaled short-acting bronchodilator given to patient for lung function testing. Long-acting bronchodilators are typically not withdrawn for clinical testing in UKSAR.

Supplemental Table S11

**Criteria**	**Ineligible**		**Eligible**		**Minimal Adjusted OR (95% CI)**	**Fully Adjusted OR (95% CI)**
	Total Number	Composite Responders (%)	Total Number	Composite Responders (%)		
Suspected comorbid COPD	152	119 (78.3%)	1109	836 (75.4%)	1.19 (0.78, 1.82)	1.12 (0.72, 1.74)
Suspected ABPA	41	31 (75.6%)	1251	947 (75.7%)	1.00 (0.47, 2.16)	1.15 (0.51, 2.58)

Suppl Table S11: Odds Ratios for composite response in eligible patients versus ineligible patients for each eligibility sensitivity analysis

Minimally adjusted models included adjustment for hospital site. Fully-adjusted models additionally included age (5-year categories), gender and pre-biologic blood eosinophil counts.

<u>Supplemental Table S12</u>

**Criteria**	**Response Domain**	**Ineligible**		**Eligible**		**Minimal Adjusted OR (95% CI)**	**Fully Adjusted OR (95% CI)**
		Total Number	Responders (%)	Total Number	Responders (%)		
Suspected comorbid COPD	ACQ Improvement ≥0.5 or Controlled	132	86 (65.2%)	879	609 (69.3%)	0.82 (0.55, 1.22)	0.79 (0.53, 1.19)
	Exacerbation Reduction >50% or No Exacerbations	153	113 (73.9%)	1112	817 (73.5%)	1.05 (0.71, 1.57)	0.99 (0.66, 1.49)
	FEV1 increase >100ml	130	59 (45.4%)	963	453 (47.0%)	0.95 (0.65, 1.38)	0.92 (0.62, 1.36)
	mOCS Dose Decrease ≥50% or No mOCS	87	43 (49.4%)	582	331 (56.9%)	0.71 (0.44, 1.15)	0.71 (0.43, 1.16)
Suspected ABPA	ACQ Improvement ≥0.5 or Controlled	32	23 (71.9%)	1004	688 (68.5%)	1.04 (0.46, 2.38)	0.96 (0.41, 2.23)
	Exacerbation Reduction >50% or No Exacerbations	41	34 (82.9%)	1254	924 (73.7%)	1.69 (0.72, 3.96)	1.63 (0.68, 3.90)
	FEV1 increase >100ml	27	12 (44.4%)	1066	499 (46.8%)	0.87 (0.39, 1.91)	0.85 (0.37, 1.91)
	mOCS Dose Decrease ≥50% or No mOCS	13	8 (61.5%)	672	371 (55.2%)	1.53 (0.44, 5.24)	1.87 (0.52, 6.67)

Suppl Table S12: Odds Ratios for domain specific responses in eligible patients versus ineligible patients for each eligibility sensitivity analysis

Minimally adjusted models included adjustment for hospital site. Fully-adjusted models additionally included age (5-year categories), gender and pre-biologic blood eosinophil counts.

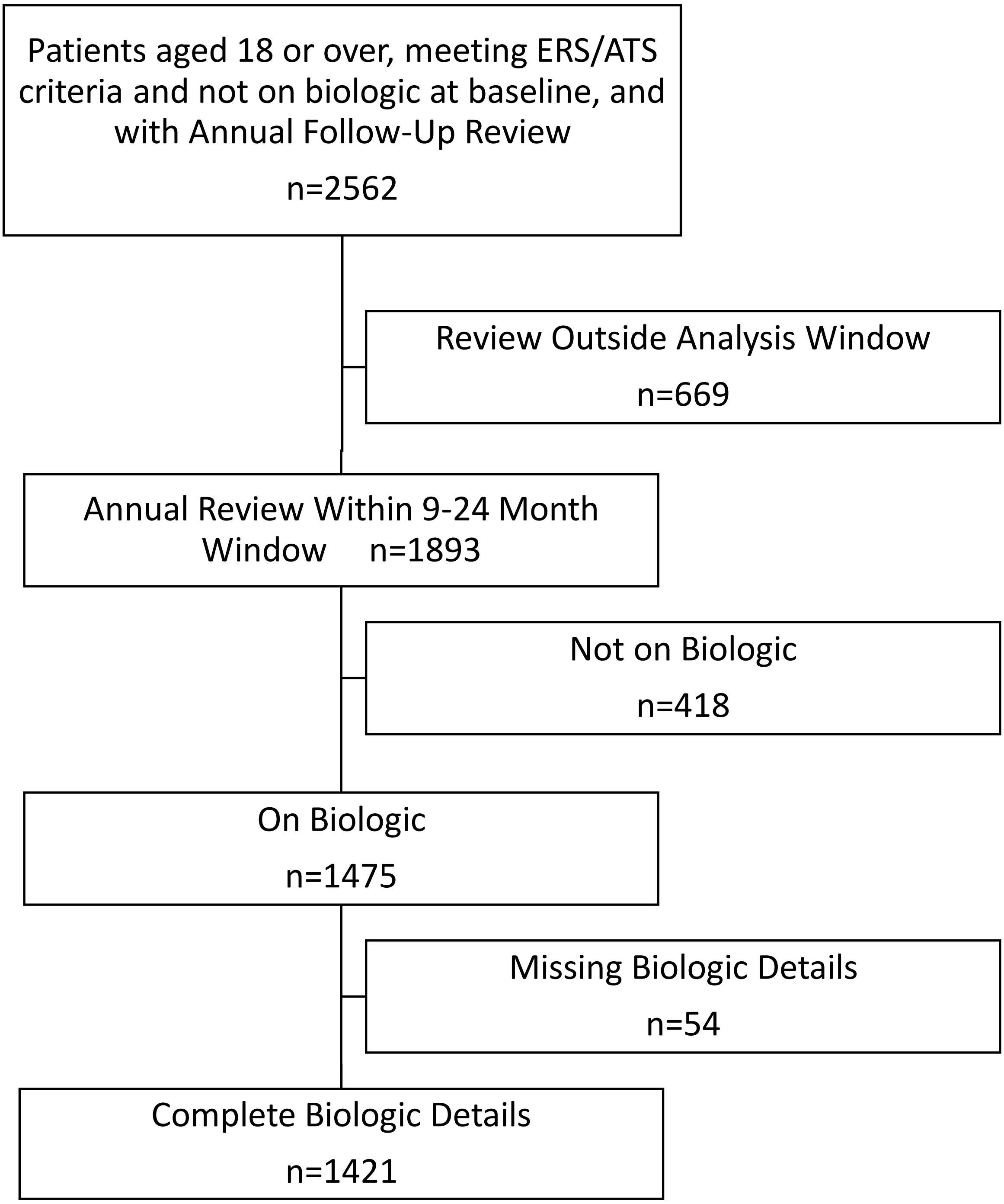

Suppl Figure S1: Consort diagram for selection of study cohort within UKSAR
